# Assessing Orthopedic Patients’ Perspectives on and Adherence to Perioperative Digital Experience Sampling

**DOI:** 10.3390/jcm14093044

**Published:** 2025-04-28

**Authors:** Jasmijn E. Willemen, Sjors C. F. van de Weijer, Richel Lousberg, Thérèse A. M. J. van Amelsvoort, Andrea J. R. Balthasar

**Affiliations:** 1 School for Mental Health and Neuroscience, Maastricht University, Universiteitssingel 40, 6229 ER Maastricht, The Netherlands; jasmijn-willemen@live.nl; 2 Center for Acute and Critical Care, MUMC+, P. Debyelaan 25, 6229 HX Maastricht, The Netherlands; 3 Department of Psychiatry and Psychology, MUMC+, P. Debyelaan 25, 6229 HX Maastricht, The Netherlands; r.lousberg@maastrichtuniversity.nl (R.L.); t.vanamelsvoort@maastrichtuniversity.nl (T.A.M.J.v.A.); 4 Department of Anaesthesiology and Pain Medicine, MUMC+, P. Debyelaan 25, 6229 HX Maastricht, The Netherlands

**Keywords:** experience sampling method, postsurgical, perioperative, acute pain, patient opinion

## Abstract

**Background/Objectives:** The experience sampling method (ESM) is a structured data collection method in which participants respond to randomly timed acoustic alerts while engaging in their daily activities. It allows patients to assess their mood, context and pain levels. This study aimed to evaluate the opinions of patients who underwent surgery on the perioperative use of a digital ESM tool. **Methods:** The Psymate app version 1.6.15 (ESM device) generated 10 short reports (acoustic alerts) at semi-random times each day for patients who underwent total knee arthroplasty (TKA). The short report, consisting of 23 items, contained questions about mood, context and pain. At the end of the study, patients’ opinions about the app were collected through a standardized questionnaire covering multiple subtopics. Feedback was analyzed using descriptive statistics. **Results:** Of the 40 patients scheduled for TKA, 31 participated in the evaluation. The app received an average user-friendliness rating of 8.3 out of 10. Some patients express dissatisfaction with the frequency (*n* = 10) and noise (*n* = 14) of the notifications. Additional analyses suggest that higher pain levels may reduce the likelihood of completing the short report. **Conclusions:** This is the first study to evaluate postoperative patients’ opinions on the use of a digital ESM app. The PsyMate app received satisfactory ratings across all subtopics, although patients identified areas for improvement.

## 1. Introduction

The experience sampling method (ESM) was developed by Prescott et al. in 1976 to integrate a systemic research approach with a more personalized perspective [1]. Related terms include ecological momentary assessment, daily diary, intensive longitudinal assessment and ambulatory assessment [2,3,4,5]. The ESM is a structured data collection method, where participants respond to randomly timed repeated acoustic alerts multiple times a day. Patients are asked the same set of questions at every acoustic alert over several days or weeks while in their natural environment [2]. This provides a comprehensive understanding of participants’ daily lives, holding more value than traditional data collection methods [6]. The ESM has several advantages: it maximizes ecological validity, making the findings more generalizable to real-world settings [7,8]. It also allows for hypothesis testing both between and within individuals and minimizes memory bias since participants report their current states directly within a limited time frame [8]. However, the ESM has limitations [6]. Some participants find it demanding, potentially resulting in a sample biased toward highly conscientious individuals [9]. Furthermore, repeatedly reporting moods can impact participants’ emotions and increase self-awareness during the data collection [10]. In medical settings, the ESM typically captures somatic, psychological and social context-related information, which are often interrelated in complex ways. Since ESM data involve repeated measures and have a multilevel structure, advanced statistical methods, such as multilevel analysis, are required, which poses challenges for both the researchers and clinicians alike [11].

In the clinic, the ESM is used with a wide range of patients, including patients with psychiatric conditions and chronic somatic disorders [6]. To our knowledge, this study is the first to evaluate a digital ESM application in a perioperative acute pain setting (e.g., patients undergoing total knee arthroplasty [TKA]). Patients received ESM questions on their phones via a specific app (e.g., PsyMate) before and after surgery, both in the hospital and home settings. Our clinical objectives in the pilot studies were to enhance the understanding of postoperative pain and the influence of various psychological factors on pain intensity and its course. In addition, through retrospective evaluations, we gathered patients’ feedback on their experience with the process. This article summarizes this feedback and presents analyses of some behavioral data concerning the response rate.

This feedback can provide deeper insights into the strengths and weaknesses of the ESM method. In recent years, patients have been discharged from the hospital earlier; thus, maintaining visibility into their postoperative pain process is essential. Acute postoperative pain is a risk factor for long-term chronic pain, making it crucial to understand patient’s postoperative pain experiences [12]. This app has the potential to become a cornerstone of personalized medicine in postoperative acute pain management. Through this evaluation, we can assess, at an early stage, whether patients are willing and able to be monitored using such a tool. Previous studies have shown positive results regarding the usability of various ESM applications in different settings. However, to our knowledge, no study has yet examined the ESM’s use in the perioperative setting, where evaluation information could be very useful. Through this evaluation, we aim to identify the positive and negative aspects of ESM to optimize its use in perioperative care. Ultimately, we seek to improve the patients’ health outcomes by translating the behavioral findings of the ESM and ascertain whether the ESM holds promise for implementation across diverse healthcare sectors.

## 2. Materials and Methods

### 2.1. Content of the Structured Interview

The interview questions were based on the system usability scale and were refined by an expert team consisting of an anesthesiologist and two health scientists. The quality and comprehensibility of the questions were further enhanced through a Delphi-like panel discussion (Supplement S1).

### 2.2. ESM Application

The Psymate app (www.psymate.eu) is designed for mobile devices (Android and iOS) and is based on the ESM method. It was developed and validated by the Department of Psychiatry and Neuropsychology at Maastricht University, Maastricht UMC+, and Smartehealth GmbH ( ECS International B.V. Walburg 12, 6225 CM Amby-Maastricht) in The Netherlands.

### 2.3. Study Design and Ethical Approval

The Psymate app was used in this prospective observational pilot study conducted between December 2017 and March 2019 at Maastricht University Medical Centre.

This evaluation study did not require direct ethical approval. The study on which the evaluation is based, where the patients used the PsyMate application, did receive ethical approval from the Medical Ethical Committee of the academic hospital Maastricht and the University of Maastricht (METC), The Netherlands. The METC number is: 2017-0145.

Participants who signed informed consent forms, those fluent in speaking and reading Dutch, those aged between 18 and 80 years, those being scheduled for TKA and those who owned and used a smartphone were included in this study.

Individuals with visual impairment, no knowledge of how to complete a digital questionnaire and those undergoing a revision of a total knee replacement were excluded. Upon inclusion, each patient received a briefing from a mobile health officer at the hospital, who explained this study and the app’s technical aspects.

The ESM questions comprised self-assessment momentary questions (short report questions) (Supplement S2). Each day, at semi-random times (ensuring a balanced spread over the day), the Psymate app generated 10 acoustic alerts to remind the patient to fill in a short report. To prevent participants from planning their day around the alerts, the unpredictability of the short report occurrence was essential. Thus, participants were requested to provide a short report of their situation up to 10 times a day between 7:30 a.m. and 10:30 p.m. The short report had to be completed within 15 min of receiving the short report to be valid. Each report consisted of the same set of 23 items with questions about mood, context, pain and physical factors, with an average completion time of 90 s. 

The ESM questionnaires (short reports) were administered for two consecutive days before surgery, at least a week before the surgery, and continued from the day after surgery until a maximum of postoperative day 6. This schedule included both hospital stays and home recovery after discharge. A 7-point numeric rating scale (NRS: 1 = none, 4 = moderate, 7 = very) was used for the questions. Upon the completion of each short report, all responses were automatically uploaded and pseudonymized to a cloud-based database. 

### 2.4. Evaluation Interview Procedure

Evaluation questions were administered through a structured telephone interview conducted by a research nurse within a week of this study’s completion. These evaluation questions covered several subtopics, including usability, subjective perceived burden, question content, technical problems, clinical implications and overall satisfaction. Some responses were rated from 1 (not at all) to 7 (very). Other questions were open-ended or required a simple “yes” or “no” answer. Supplement 3 shows a free translation of all answers to the open-ended questions. For one question (how the app was rated overall), participants rated the question on a scale in the range of 0–10.

### 2.5. Surgical Procedure: TKA

Patients were electively scheduled for TKA after anesthesiologic approval. The hospital protocol allowed for surgery under general or spinal anesthesia. Typically, patients for this procedure are usually discharged approximately 2 days later.

Thus, the PsyMate application was used before surgery at home, after knee surgery in the hospital and during the first few days following discharge. 

### 2.6. Statistical Analysis

Responses from the evaluation questionnaire were analyzed using descriptive statistics and divided into subtopics. Depending on the response options, either the mean and standard deviation were calculated, or the percentage for “yes” or “no” responses was reported. Answers to open-ended questions were reviewed and included in the discussion. Due to the aim of this evaluation and the sample size, we decided to not apply inferential statistics.

### 2.7. ESM Response Rate

Additional analyses were conducted on the ESM response rate. As a secondary objective, we analyzed the relation between time and pain compared to the response rate of the short reports.

## 3. Results

Forty patients were scheduled for TKA, all of whom were approached for the evaluation. Of these, 31 participated in the evaluation process with a final valid evaluation. The participants had a mean age of 60.9 ± 8.7 years, and the average hospital stay was 2.3 ± 0.8 days. The group consisted of 12 male and 19 female patients. An additional nine patients completed the full protocol but were not participating in the evaluation. This group had a mean age of 64.2 ± 8.7 years (*p* = 0.33) and consisted of six male and three female participants (*p* = 0.46); both were not significantly different between the responder and non-responder groups.

The answers were categorized into subtopics.

### 3.1. Usability Evaluation

Overall, participants found the PsyMate app easy to use (Table 1). Most participants (96.7%) had the opinion that the text on the app screen was easy to read. The app was also easy to operate, with 87.1% and 90.0% of participants reporting no difficulty in starting the app or navigating its interface. Both oral and written instructions were deemed clear, scoring 6.9 ± 0.3 and 6.5 ± 1.2, respectively, on a 7-point NRS.

### 3.2. Subjectively Perceived Burden

Areas for improvement were noted in the subcategory of perceived burden. The burden related to the number of alerts was rated moderate (3.1 ± 2.0), as well as the noise of notifications (3.4 ± 2.0). However, participants indicated less burden related to the duration of the questions (1.8 ± 1.5), with 64.5% strongly disagreeing that the question length was burdensome (Table 2).

### 3.3. Content of the Questions

The final question, “What did you think of the content of the questions?”, had five response options: adequate, unnecessary, missed, redundant or annoying. Participants found the questions somewhat difficult or unclear, with a mean score of 2.6 ± 2.2. Most participants (58.1%) strongly disagreed that the questions were difficult, while 19.4% slightly agreed and 3.2% strongly agreed. In addition, during the debriefing, 90.3% of the participants said the content of the questions was “adequate”, whereas 6.5% and 3.2% of the participants found some questions “redundant” or “annoying”, respectively. Owing to different response scales, these results are not shown in Table 3. 

### 3.4. Technical Problems

Technical issues did not significantly hinder question completion, with a mean score of 1.8 ± 1.8. Most participants (77.4%) strongly disagreed that technical problems prevented them from completing the questions properly. Those who did encounter problems mentioned difficulties logging in or occasional app crashes in the open-ended responses. The results for this subtopic are shown in Table 4.

### 3.5. Impact on Daily Life

The study had a minimal impact (Table 5). Participants reported that their mood, activities, contact with others and daily routines were largely unaffected, with all categories showing low mean scores. Specifically, the mean scores for impact on mood (1.4 ± 1.0), activities (1.4 ± 0.8), contact with others (1.4 ± 0.7) and daily activities (1.3 ± 0.5) indicate that participation did not significantly disrupt their daily lives.

### 3.6. Relevance for Care Practice

The yes/no questions in this subtopic underscore significant clinical implications for integrating digital health tools in patient care. Most participants (93.5%) preferred their anesthesiologist or pain nurse to access their records for additional information or advice, and nearly all (96.8%) found immediate digital advice regarding pain medication helpful. Furthermore, over half (54.8%) valued having access to their pain and mood data during the measurement period.

### 3.7. Overall Satisfaction

Importantly, in terms of overall satisfaction, all participants expressed a willingness to use the ESM application again for future surgeries, rating its overall usability highly, with an average score of 8.4 ± 0.7 out of 10.

### 3.8. Analysis of ESM Response Rate

**Figure 1:**
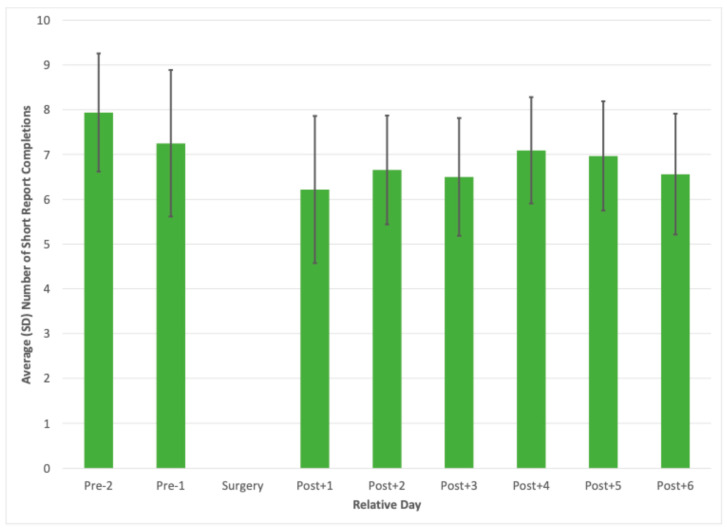
Average number of short report completions per relative day from two days before surgery until day six after surgery.

As illustrated in Figure 1, the daily number of short reports completed pre-surgery is higher than those completed post-surgery, indicating greater subject participation prior to the surgery. The average number of short reports completed on Post + 1 was relatively (still adequate) low, with an average of 5.8 ± 3.6, followed by a slight increase in Post + 2. Subsequently, the number of completions stabilized and increased slightly toward Post + 4 and Post + 5 before experiencing a modest decline in Post + 6. Overall, the data indicate a gradual increase in the number of short reports completed over time post-surgery, suggesting improved participant engagement as recovery progressed.

**Figure 2:**
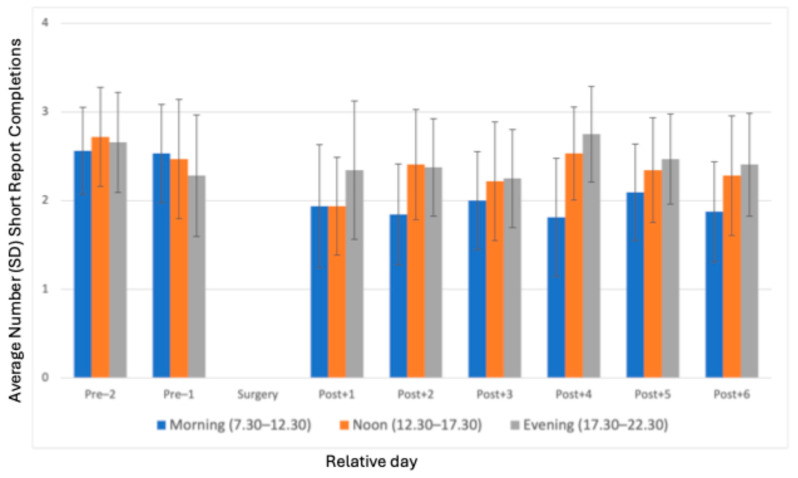
Average number of short report completions per morning/noon/evening from two days before surgery until day six after surgery.

A breakdown by specific periods—morning (7:30 a.m.–12:30 p.m.), noon (12:30 p.m.–5:30 p.m.) and evening (5:30 p.m.–10:30 p.m.)—provides additional insights, as depicted in Figure 2. Throughout the study, evening completions consistently outnumbered those in the morning and noon. On Post + 1, evening completions averaged 2.3 ± 1.6 short reports, while morning and noon completions averaged approximately 1.9 ± 1.4 and 1.9 ± 1.1, respectively. This pattern continued on subsequent days, with evening sessions typically exhibiting the highest engagement, followed by noon and morning sessions.

**Figure 3:**
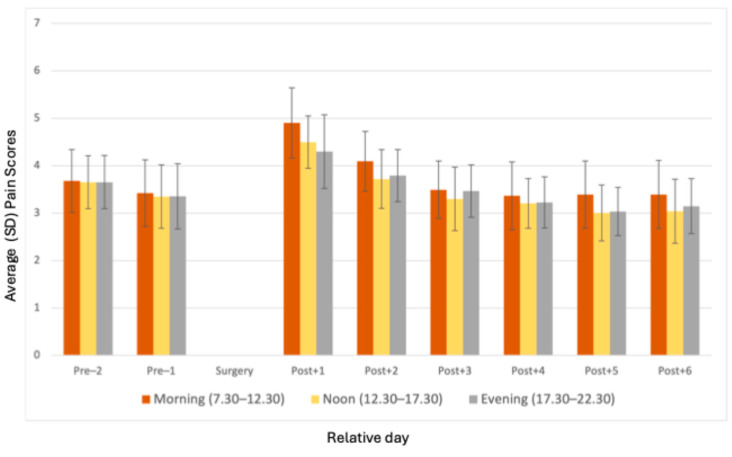
Average pain scores per morning/noon/evening from two days before surgery until day six after surgery.

Moreover, a visual inspection of the data (Figure 3) indicates a negative correlation between pain levels and the frequency of completed short reports across different times of the day (morning, noon and evening) following surgery. This indicates that higher pain levels may reduce the likelihood of participants completing short reports. Interestingly, pain scores were higher in the mornings, gradually decreasing throughout the day. This reduction in pain during the noon and evening periods was associated with an increase in the number of short reports filled out, particularly in the evening.

## 4. Discussion

This study is one of the first to use digital ESM in a perioperative setting, specifically assessing patients’ experiences with the PsyMate app following TKA. The findings from this study can guide improvements in future studies using this method. Multiple subtopics were evaluated to assess the strengths and weaknesses of the PsyMate app, a mobile app using the ESM method. Following app usage for almost 1.5 weeks, participants provided an evaluation of various subtopics, including usability, content and overall satisfaction.

Overall, the app received positive ratings and had no significant impact on daily activities, emotions or social interactions. All participants expressed a willingness to use the app again in future surgeries, highlighting its potential for perioperative care integration. Most participants also wanted healthcare providers to access these data for postoperative advice.

Participants were generally satisfied with the content of the questions; however, some noted a lack of answer options and a need for more open-ended questions. Addressing these minor points can meet the needs of the patients and improve the integration of this digital tool in perioperative care. A notable challenge identified was the perceived burden of frequent acoustic alerts and notification sounds (e.g., the notification melody). The average burden ratings were relatively low (3.1–3.4 on a 7-point NRS); some participants indicated that receiving 10 acoustic alerts per day or having alerts occur too closely together was too much. The results align with the results from earlier studies evaluating ESM applications. In patients with chronic diseases, including small-fiber neuropathy, Damci et al. reported similar positive outcomes on the acceptability, usability and feasibility of the ESM method [13]. Some participants in our study found the number of acoustic alerts burdensome, consistent with the observations from previous studies, such as that of Vork et al. [14], which noted that the ESM is time-consuming and can lead to reduced compliance. However, the repetitive nature of the ESM is intentional, as it reduces the impact of missing data on the overall results.

In our study, the daily response rate was fairly good, with most postoperative days having an average of at least 60% completed short reports. In comparison, other studies reported similar response rates of 50–70% in target groups of transdiagnostic outpatients and patients with acquired brain injury [15,16]. Looking at the raw data, our data reveal a negative correlation between pain levels and the ESM completion rate, which are generally lower when a person has higher pain levels. In addition, there was a significant difference between the response date Pre-2 and Post+1. Indeed, the number of completed short reports was the lowest on the first postoperative day, with an average of 6.2 out of 10 per day. The average of completed short reports slightly increased in subsequent days. The changes were subtle, and the adherence levels remained high throughout the whole study period.

Adherence also varied by time of day, with the evening slot being the most preferred or convenient time for questionnaire completion, suggesting that patients had more time to respond to alerts in the evening or because postoperative pain was at its lowest during this time slot, as concluded by a previous analysis from this study [17]. These findings suggest that physical (postoperative) pain interacts with an adherence to an ESM application. Time-of-day effects should be considered when designing ESM data collection protocols to maximize the response rates. Our data indicate that higher pain levels may reduce the likelihood of participants completing short reports (Figure 2 and Figure 3).

This correlation between higher pain scores and short report non-completion represents a classic type of non-random missing-data pattern, in which studies interested in post-surgical pain are likely to systematically underestimate patients’ actual pain levels, because the times at which pain is greatest are also precisely those times at which a survey is most likely to be skipped. For future studies, it could be interesting to adjust for this relationship in interpretation by adding some inflation factor to the pain scores that are reported on any given day.

The target group’s characteristics may influence adherence to ESM protocols, as certain populations may find it more challenging to comply with frequent assessments, potentially influencing the method’s feasibility and reliability. Van Genugten et al. found that participants with affective disorders experienced more burden in ESM studies from mental well-being questions compared to healthy participants after 2 weeks of the ESM [18]. However, these patients also showed high adherence. Given that the present study lasted only half as long, it suggests that applying shorter studies using ESM questions is feasible.

However, this study had some limitations. First, there might have been selection bias, as the sample consisted only of patients with TKA, predominantly from a specific age group, who were likely frequent mobile phone users. This limited the generalizability of the findings to other surgical populations or patients less comfortable with technology. Second, the evaluation method—conducted via one-on-one telephone interviews—might have constrained the depth of feedback due to the absence of non-verbal cues and participants feeling reluctant to criticize the application in a direct conversation openly.

Technological advancements have already improved the ESM; however, they also present future challenges, such as battery optimization (e.g., energy consumption management settings that prevent apps from running in the background) [19]. Conversely, these advances may also create new opportunities. For example, mobile context awareness can play a larger role in the ESM, with mobile devices perceiving and responding to contextual cues (e.g., a smartphone with location-based notification via GPS or combined activity/health data gathered by a smartwatch). In the future, multiple devices and networks could integrate, enabling a deeper understanding of an individual’s context, behavior and experiences [19]. All this together suggests that the ESM can play a valuable role in personalized healthcare, enabling professionals to respond in real time to patients’ pain or behavioral patterns when necessary. The ESM can bridge the gap between healthcare professionals and personalized perioperative care in the home setting. This may indicate that patients are not only open to digital monitoring, but also value interactive, personalized care during the postoperative period. Caregivers can respond to monitored and recorded behaviors or pain when appropriate. In addition to providing direct recommendations, the ESM can also be employed to create personalized pathways for (pain) medication initiation or discontinuation. Analyzing the ESM data to map individual pain profiles can help identify patients at higher risk of developing chronic pain. By implementing targeted interventions, the risk of transitioning to chronic pain can be diminished. When a patient’s recovery is at risk, the ESM can facilitate direct consultations through the app, lowering the threshold for patients to contact healthcare professionals. Furthermore, certain patterns in a person’s disease can be detected, enhancing diagnosis and treatment [15]. Yu et al. discussed the potential for the ESM in hospitality management research, which can serve as a starting point for the use of the ESM in diverse sectors [20].

## 5. Conclusions

In conclusion, the ESM app Psymate received positive ratings across all evaluated subtopics, demonstrating high acceptance among postoperative patients. All participants expressed a willingness to use the app again, with an overall user-friendliness rating of 8.3 out of 10. The high adherence rates and willingness to reuse the app suggest its potential for future integration into perioperative care.

However, this study also highlighted areas for improvement, particularly in notification management and direct health information provision. Further research is needed to validate these findings and evaluate the impact of addressing these challenges. While the ESM may present challenges in the future, it also holds promising opportunities, particularly for improving patient care across various sectors.

## Figures and Tables

**Table 1:** Usability evaluation results (*n* = 31, 7-point numeric rating scale). Darker shades of orange indicate relatively higher percentages, while lighter shades reflect less frequently occurring values.

Question	Mean (SD)	Strongly Disagree	Disagree	Slightly Disagree	Neither Agree nor Disagree	Slightly Agree	Agree	Strongly Agree
Could you easily read the texts on the screen?	6.6 (0.6)	0.0%	0.0%	0.0%	0.0%	3.2%	29.0%	67.7%
Did you find it difficult to turn on the app?	1.4 (1, 4)	87.1%	6.5%	0.0%	0.0%	0.0%	3.2%	3.2%
Did you find it difficult to operate the app?	1.4 (1.4)	90.0%	3.3%	0.0%	0.0%	0.0%	3.3%	3.3%
Was the verbal explanation you received about using the app clear?	6.9 (0.3)	0.0%	0.0%	0.0%	0.0%	0.0%	12.9%	87.1%
Was the written explanation you received with the app clear?	6.5 (1.2)	3.2%	0.0%	0.0%	0.0%	3.2%	22.6%	71.0%

**Table 2:** Results of the subtopic “subjectively perceived burden”. Darker shades of orange indicate relatively higher percentages, while lighter shades reflect less frequently occurring values.

Question	Mean (SD)	Strongly Disagree	Disagree	Slightly Disagree	Neither Agree nor Disagree	Slightly Agree	Agree	Strongly Agree
Did you find using the app burdensome in terms of the number of beeps per day?	3.1 (2)	29.0%	19.4%	19.4%	6.5%	9.7%	6.5%	9.7%
Did you find using the app burdensome in terms of duration of the questions?	1.8 (1.5)	64.5%	22.6%	3.2%	0.0%	0.0%	9.7%	0.0%
Did you find using the app burdensome in terms of noise?	3.4 (2)	29.0%	9.7%	16.1%	16.1%	6.5%	16.1%	6.5%

**Table 3:** Results of the subtopic “content of the questions”. Darker shades of orange indicate relatively higher percentages, while lighter shades reflect less frequently occurring values.

Question	Mean (SD)	Strongly Disagree	Disagree	Slightly Disagree	Neither Agree nor Disagree	Slightly Agree	Agree	Strongly Agree
Were the questions from the app difficult or unclear?	2.6 (2.2)	58.1%	9.7%	0.0%	3.2%	6.5%	19.4%	3.2%
Do you feel that you were able to accurately convey your experiences with the questions?	5.9 (1)	0.0%	0.0%	3.2%	6.5%	12.9%	51.6%	25.8%

**Table 4:** Results of the subtopic “technical problems”. Darker shades of orange indicate relatively higher percentages, while lighter shades reflect less frequently occurring values.

Question	Mean (SD)	Strongly Disagree	Disagree	Slightly Disagree	Neither Agree nor Disagree	Slightly Agree	Agree	Strongly Agree
Did (technical) problems prevent you from completing the questions properly?	1.8 (1.8)	77.4%	6.5%	0.0%	3.2%	3.2%	6.5%	3.2%

**Table 5:** Results of the subtopic “clinical implications”. Darker shades of orange indicate relatively higher percentages, while lighter shades reflect less frequently occurring values.

Question	Mean (SD)	Strongly Disagree	Disagree	Slightly Disagree	Neither Agree nor Disagree	Slightly Agree	Agree	Strongly Agree
Did participating affect your mood?	1.4 (1)	77.4%	12.9%	3.2%	3.2%	3.2%	0.0%	0.0%
Did participating affect your activities?	1.4 (0.8)	77.4%	12.9%	6.5%	3.2%	0.0%	0.0%	0.0%
Did participating affect your contact with others?	1.4 (0.7)	71.0%	19.4%	9.7%	0.0%	0.0%	0.0%	0.0%
Did participating hinder you in your daily activities?	1.3 (0.5)	71.0%	25.8%	3.2%	0.0%	0.0%	0.0%	0.0%

## Data Availability

Research data are available upon request from the corresponding authors.

## Supplementary Materials

The following supporting information can be downloaded at: https://www.mdpi.com/article/10.3390/jcm14093044/s1. Supplement S1: Evaluation questions; Supplement S2: ESM questions (short reports); Supplement S3: All patient answers to the open questions.

## Abbreviations

The following abbreviations are used in this manuscript:

ESM	Experience sampling method
TKA	Total knee arthroplasty
NRS	Numeric rating scale
