# Supplementary material for: Assessing Orthopedic Patients’ Perspectives on and Adherence to Perioperative Digital Experience Sampling
Source: J Clin Med. 2025 Apr 28;14(9):3044. doi: 10.3390/jcm14093044 (PMC12072669; doi:10.3390/jcm14093044)
Supplement: Supplementary file 1 [file jcm-14-03044-s001.zip › jcm-3522718-supplementary.pdf]

Supplement 1: Evaluation questions

**ESM protocol: evaluation questions**			
**Domain**	**Item**	**Description**	**Scale**
**Research?**	1	Did you find this to be a typical week?	1=not, 4=moderate, 7=very
	2	Did special events play a role this week?	1=not, 4=moderate, 7=very
	2a	If yes, which ones?	
	3	Do you feel that you were able to accurately represent your experiences in the questions that were asked?	1=not, 4=moderate, 7=very
	4	Did participating affect your mood?	1=not, 4=moderate, 7=very
	5	Did participating affect your activities?	1=not, 4=moderate, 7=very
	6	Did participating affect your contact with others?	1=not, 4=moderate, 7=very
	7	Did participating interfere with your daily activities?	1=not, 4=moderate, 7=very
	8	Did you often make mistakes while filling in the answers?	1=not, 4=moderate, 7=very
	8a	If yes, how often and with which questions?	

	9	Do you have any other comments?	
**Usability?**	1	Could you read the text on the app's screen clearly?	1=not, 4=moderate, 7=very
	2	Did you find it difficult to turn on the app?	1=not, 4=moderate, 7=very
	3	Did you find it difficult to operate the app?	1=not, 4=moderate, 7=very
	4	Was the verbal explanation you received about using the app clear?	1=not, 4=moderate, 7=very
	5	Was the written explanation clear?	1=not, 4=moderate, 7=very
	6	Were the questions from the app difficult or unclear?	1=not, 4=moderate, 7=very
	6a	If yes, which ones?	1=not, 4=moderate, 7=very
	7a	Did you find the use of the app burdensome in terms of number ofshort reports per day?	1=not, 4=moderate, 7=very
	7b	Did you find the use of the app burdensome in terms of duration of the questions per short report?	1=not, 4=moderate, 7=very

	7c	Did you find the use of app burdensome in terms of the sound?	1=not, 4=moderate, 7=very
	8	Did (technical) problems prevent you from answering the questions?	1=not, 4=moderate, 7=very
	8a	If yes, which ones?	
	9	Any other comments?	
**Debriefing**	1	To what extent have you recovered after the surgery, from 0–100%?	Score 0–100%
	2	Evaluation of the surgery's effect	1=very disappointing, 2=disappointing, 3=neutral, 4=satisfactory, 5=very satisfactory
	3	Was the accompanying information clear and sufficient?	1=yes, 0=no
	9	Was the researcher available for questions during the study?	1=yes, 0=no
	4	Did you find it burdensome to carry the iPod/phone with you all day?	1=yes, 0=no

	5	How much time did it take on average to complete one short report?	1 = 0–1 min, 2 = 1–2 min, 3 = 2–5 min, 4 = >5 min
	6a	Did you miss or fail to answer any short reports?	1=yes, 0=no
	6b	If yes, approximately how often?	1=rarely, 2=sometimes, 3=regularly, 4=often, 5=very often
	6c	What was the reason?	1=did not hear, 2=forget, 3=no time
	7a	Do you have suggestions regarding the questions?	1=yes, 0=no
	7b	Number of short reports	1=too few, 4=just right, 7=too many
	7c	What did you think of the content of the PsyMate questions?	1=adequate, 2=unclear questions, 3=missed questions, 4=unnecessary questions, 5=annoying questions
	8	Would you like to use the PsyMate again for a future surgery?	1=yes, 0=no

	9	Would you like to have access to your pain and mood progress during the measurement period?	1=yes, 0=no
	10	Would you find it useful to receive immediate digital advice while answering the questions, for example, regarding pain medication?	1=yes, 0=no
	11	Would you like your anesthesiologist or pain nurse to view your data so that they can call to provide additional information/advice if necessary?	1=yes, 0=no
	12	Give a score (between 0–10) for the overall user-friendliness of the Psymate	Score 0–10

Supplement 2: ESM questions (short reports)

**ESM protocol: short report questions (10 per day)**			
**Domain**	**Item**	**Description**	**Scale**
**Mood**	1	I feel cheerful	1=not, 4=moderate, 7=very
	2	I feel irritated	1=not, 4=moderate, 7=very
	3	I feel relaxed	1=not, 4=moderate, 7=very
	4	I feel lonely	1=not, 4=moderate, 7=very
	5	I feel anxious	1=not, 4=moderate, 7=very
	6	I feel satisfied	1=not, 4=moderate, 7=very
	7	I feel sad	1=not, 4=moderate, 7=very
	8	I feel guilty	1=not, 4=moderate, 7=very
	9	I feel confident	1=not, 4=moderate, 7=very
	10	I feel stressed	1=not, 4=moderate, 7=very
	11	I feel worried	1=not, 4=moderate, 7=very
	12	Overall I feel good	1=not at all, 4=moderately, 7=very
**Context**	13	Physical activity: I…	Lie down / sit / stand / walk / cycle / exercise / other

	14	What are you doing?	TV watching / reading / music listening / internet, social media / telephone / resting / working / household / hygiene / eating, drinking / talking, having a conversation / something else
	15	I do this for	My recovery / my general health / fun / distraction / because I must / for my work / my future / others / other
	16	This is hard for me	1=not, 4=moderate, 7=very
	17	I would rather do something different	1=not, 4=moderate, 7=very
	18	Where are you?	At home / at someone home / at work, school / healthcare institution / public opportunity / on the way
	19	With whom are you?	No one, alone / partner / family resident / family living away from home / friends / colleagues / acquaintances / strangers
	20	I find this pleasant	1=not, 4=moderate, 7=very
**Pain**	21	What is your pain right now?	1=not, 4=moderate, 7=very (at 1 by Unpleasant question 23)
	22	Location of the pain	1=area of operation, 2=other

	23	Did you take any pain medication since the last short report?	No / heat / cooling / (physio) exercises / paracetamol / ibuprofen or naproxen or diclofenac (Voltaren) / tramadol or zaldiar / oxycodone / alcohol / cannabis / other, namely
**Physically**	24	I am tired	1=not, 4=moderate, 7=very
	25	I suffer from	None of these / drowsiness / nausea / vomiting / itching / dizzy / short of breath / palpitations / ringing in the ears / headache / stomach ache / muscle pain / difficult bowel movements
	26	This short report bothered me	1=not, 4=moderate, 7=very
			**Thank you**

Supplement 3: open-ended questions and the responses

Domain; Research

Question 2a: Have any special events played a role this week? If so, which ones?

"Unclear."
"Surgery."
"Lawsuit and surgery."
"A lot of pain."

Question 8a: Did you often make mistakes while filling out? If so, which ones?

"I wanted to fill it out too quickly."
"Just read too quickly."
"Read too quickly."
"Made 1 or 2 mistakes."
"A few times, especially at the beginning."
"Looked back 1 or 2 times."
"Rarely, only with emotions because it was on autopilot."
"Sometimes too quickly."
"Rarely, was able to correct it well."
"Made mistakes about 10 times on different questions."
"Pressed too quickly, but was able to correct it."
"Was able to correct it, just pressed too quickly."
"A few times."

Question 9: Other comments

"I could talk about it positively with others, and I would like more options for 'What have you done for the pain?'"
"1. Physiotherapy is not listed under 'Who are you with?' 2. Foot is not listed under pain options."
"N/A"
"The sound is annoying."
"Friendly staff, everything was clear."
"The question 'Do you have pain?' was too limited. Couldn't indicate fluid retention."
"Everything was good."
"Sometimes it was annoying during an exciting movie or similar."
"The number of beeps per day is too much."
"Everything went fine."
"A week later might be too short. I only started feeling better now."
"1. 'Who are you with?' is missing a fellow patient. 2. Sometimes no list in the evening, but a beep. 3. Switching to a different pain scale is confusing (0-10/0-7)."
"'Where do you have pain?' Should include the surgical area or others. I'd like to indicate multiple pain areas at once."
"I find it annoying to fill this out during physiotherapy. Sometimes I also had too much pain to fill it out."
"I was given incorrect information at home. This was resolved on-site."
"10 is too much."
"The questions were good, but unfortunately not all questionnaires worked."

Domain; Usability

Question 6a: Were the questions in the app difficult or unclear? If yes, which ones?
"It was clear. Switching was a bit of a change at first."
"I prefer doing something else."
"I don't feel guilty, so it's a strange question."
"I miss an open-ended question."

Question 8a: Did (technical) problems prevent you from answering the questions correctly? If yes, which ones?
"Tried opening the app, 2. The app still occasionally makes sounds."
"Didn't fully complete the one-time questionnaire. Logged in again on Day 2."
"The day after stopping, I received many beeps."
"Logged in and out. The app did this on its own, I think."
"The app froze in the last 4 days, so I deleted it."
"Couldn't log in after the update."

Question 9: Other comments?
"Everything was fine, except for the multiple-choice question on the morning list."
"The written instructions contained a lot of information and repetition. Also, I found 10:30 PM too late, and I had to wait for a beep."
"N/A."
"Otherwise, the app was fine."
"The sound was noticeable!"
"It was quite burdensome, sometimes the beeps were too close together."
"It's a pity there are no open-ended questions. I would have liked to leave a comment at the end of the day."
"In the hospital, it was burdensome, but at home, it wasn't. The beeps were bothersome during visits."
"Everything went fine, but an advisory role in terms of pain management would be an improvement."
"Missed open-ended questions."
"No more than a week of filling it out. I already felt it was enough."
"Didn't miss any lists."
"I missed a few because I sleep late in the mornings."
"For 'What did you take for the pain?' I made a mistake. I only entered oxycodone for the last few days and not for the first days because I didn't see it (actually took more oxycodone)."
"A 'sleeping' button, and no more beeps afterward, is a good idea."
"It wasn't clear whether I was supposed to send it or not. No beeps when it's on the charger."

Domain; Debriefing
Question 1: Other comments?
"I find getting advice too personal, not via the app."
"I would like more open-ended questions."
"Too many psychological questions."
"Missed beeps due to technical problems."
